# Supplementary material for: Hybrid Iterating-Averaging Low Photon Budget Gabor Holographic Microscopy
Source: ACS Photonics. 2025 Jan 10;12(4):1771–82. doi: 10.1021/acsphotonics.4c01863 (PMC12007103; doi:10.1021/acsphotonics.4c01863)
Supplement: Supplementary file 1 — ph4c01863_si_001.pdf [file ph4c01863_si_001.pdf]

Hybrid iterating-averaging low photon budget Gabor holographic microscopy: Supplementary Document 1

Mikołaj Rogalski[1,*], Piotr Arcab[1], Emilia Wdowiak[1], José Ángel Picazo-Bueno[2,3], Vicente Micó[2], Michał Józwik[1], Maciej Trusiak[1,^]

[1]Institute of Micromechanics and Photonics, Warsaw University of Technology, 8 Sw. A. Boboli St., 02-525 Warsaw, Poland

[2]Departamento de Óptica y Optometría y Ciencias de la Visión, Universidad de Valencia, C/Doctor Moliner 50, 46100 Burjassot, Spain

[3]Biomedical Technology Center, University of Muenster, Mendelstr. 17 D-48149, Muenster, Germany

*mikolaj.rogalski.dokt@pw.edu.pl

^maciej.trusiak@pw.edu.pl

Pages S1-S8, Figures S1-S7

1. Gabor averaging method

The workflow of the Gabor averaging (GA) algorithm is illustrated in Fig. S1. The GA algorithm takes as input N in-line holograms, where each hologram H_n $(n = 1, 2, \ldots, N)$ corresponds to a unique defocus distance (z_n) or wavelength (λ_n). These holograms are then backpropagated numerically to the sample plane using the angular spectrum (AS) propagation method.[1,2] This technique reconstructs the complex optical field R_n for each hologram, representing the amplitude and phase of the sample's wavefront.

For wavelength-multiplexed datasets, the phase component φ_n of each reconstructed wavefront is rescaled by a factor of λ_n/λ_1, aligning the phase of the n-th hologram to that of the first hologram, H_1, to ensure consistent phase across varying wavelengths. To correctly transition from the intensity to amplitude domain, the amplitude part of R_n ($|R_n|$) is transformed by taking the square root of its values.

In the final step, all N complex fields R_n are averaged to produce the final Gabor-averaged reconstruction (R). This averaging process mitigates the adverse effects of each piece of information that is inconsistent in reconstructed complex fields, i.e. shot noise, coherent noise and, partially, twin image disturbance.

Algorithm S1 – GA method

Inputs: $H_n, \lambda_n, z_n \quad (n = 1,2, \ldots, N)$
Output: R

$R = 0$
for $n = 1:N$
  $R_n = \mathrm{AS}(H_n, \lambda_n, -z_n)$ % Propagate hologram to object plane
  $R = R + \sqrt{|R_n|} e^{i\varphi \frac{\lambda_n}{\lambda_1}}$ % $\varphi = \tan^{-1}(imag(R_n)/real(R_n))$
end
$R = R/N$

Fig. S1. The workflow of Gabor averaging method.

2. Gerchberg-Saxton method (multi-height)

Figure S2 illustrates the workflow of the iterative multi-height Gerchberg-Saxton (GS) algorithm.[3–6] This method processes N in-line holograms H_n, each recorded at a distinct defocus distance z_n while using the same wavelength λ.

The algorithm begins by estimating the initial complex optical field (U_1) at the first hologram plane as the square root of first hologram (with phase factor equal 0). Then, the iterative procedure starts, in which the optical field at the n-th hologram plane U_n is numerically propagated to the $n + 1$ plane, using the angular spectrum method (over the propagation distance $z_{n+1} - z_n$). Next, the U_{n+1} field is updated by replacing its amplitude with the square root of the H_{n+1} hologram, while preserving the phase from the propagated field.

Abovementioned operations are repeated for all subsequent holograms until the optical field reaches the last hologram plane U_N. After updating the field at this plane, it is backpropagated to the first hologram plane, where the amplitude is once again updated using the first hologram H_1.

The entire process is repeated for a user-defined number of iterations T, during which the phase information is refined with each cycle, converging toward the true phase distribution. After the

final iteration, the retrieved complex optical field at the first hologram plane U_1 is backpropagated to the sample plane, yielding the sample's amplitude and phase reconstructions (R).

Algorithm S2 – GS method (muti-height)

Inputs: $H_n, \lambda, z_n, T \quad (n = 1,2, \ldots, N)$
Output: R

1. $U_1 = \sqrt{H_1}$ % Initialize optical field in first hologram plane
2. **for** $t = 1{:}T$
3. **for** $n = 1{:}(N-1)$
4. $U_{n+1} = \mathrm{AS}(U_n, \lambda, z_{n+1} - z_n)$ % Propagate optical field to n+1 plane
5. $U_{n+1} = U_{n+1}\frac{\sqrt{H_{n+1}}}{|U_{n+1}|}$ % Actualize optical field with n+1 hologram
6. **end**
7. $U_1 = \mathrm{AS}(U_N, \lambda, z_1 - z_N)$ % Propagate optical field to 1st plane
8. $U_1 = U_1\frac{\sqrt{H_1}}{|U_1|}$ % Actualize optical field with 1st hologram
9. **end**
10. R =AS$(U_1, \lambda, -z_1)$ % Backpropagate optical field to the sample plane

Fig. S2. The workflow of multi-height Gerchberg-Saxton method.

3. Gerchberg-Saxton method (multi-wavelength)

Figure S3 illustrates the workflow of the multi-wavelength GS algorithm.[7–9] This approach builds upon the traditional multi-height GS method but introduces an additional layer of complexity by exploiting wavelength diversity for enhanced phase reconstruction.

In the multi-wavelength GS algorithm, N holograms H_n are acquired at varying wavelengths λ_n (and optionally defocus distances z_n), each hologram providing distinct optical information due to the wavelength-dependent diffraction nature of light. The reconstruction process begins similarly to the multi-height GS method by estimating the initial complex optical field U_1 at the first hologram plane, where the amplitude is approximated from the square root of the hologram intensity, and the initial phase is set to zero.

The primary distinction between the multi-wavelength and multi-height approaches lies in how the optical field is propagated between the hologram planes. In the multi-height version, the U_n optical field was propagated directly to the $n+1$ plane, whereas in the multi-wavelength version, the optical field U_n is firstly numerically propagated to the sample plane (with the wavelength λ_n). At the sample plane, the phase of the field (R_n) is rescaled by the λ_n/λ_{n+1} factor to account for the change in wavelength between adjacent holograms. Then the R_n is propagated to the $n+1$ hologram plane (with the wavelength λ_{n+1}). This process of propagation, phase rescaling, and backpropagation is repeated for all subsequent holograms, ensuring the accurate combination of phase information across multiple wavelengths.

Algorithm S3 – GS method (muti-wavelength)

Inputs: $H_n, \lambda_n, z_n, T \quad (n = 1,2, \dots, N)$
Output: R

1. $U_1 = \sqrt{H_1}$ % Initialize optical field in first hologram plane
2. **for** $t = 1{:}T$
3. **for** $n = 1{:}(N-1)$
4. $R_n = \mathrm{AS}(U_n, \lambda_n, -z_n)$ % Backpropagate optical field to sample plane
5. $R_n = |R_n| e^{i\varphi \frac{\lambda_n}{\lambda_{n+1}}}$ % Rescale phase; $\varphi = \tan^{-1}(imag(R_n)/real(R_n))$
6. $U_{n+1} = \mathrm{AS}(R_n, \lambda_{n+1}, z_{n+1})$ % Propagate optical field to n+1 plane
7. $U_{n+1} = U_{n+1} \frac{\sqrt{H_{n+1}}}{|U_{n+1}|}$ % Actualize optical field with n+1 hologram
8. **end**
9. $R_N = \mathrm{AS}(U_N, \lambda_N, -z_N)$ % Backpropagate optical field to sample plane
10. $R_N = |R_N| e^{i\varphi \frac{\lambda_N}{\lambda_1}}$ % Rescale phase; $\varphi = \tan^{-1}(imag(R_n)/real(R_n))$
11. $U_1 = \mathrm{AS}(R_N, \lambda_1, z_1)$ % Propagate optical field to 1st plane
12. $U_1 = U_1 \frac{\sqrt{H_1}}{|U_1|}$ % Actualize optical field with 1st hologram
13. **end**
14. $R = \mathrm{AS}(U_1, \lambda_1, -z_1)$ % Backpropagate optical field to the sample plane

Fig. S3. The workflow of multi-wavelength Gerchberg-Saxton method.

4. Iterative Gabor averaging (IGA) method pseudo-code

Figure S4 illustrates the workflow of the proposed Iterative Gabor Averaging (IGA) algorithm. The algorithm starts by performing two parallel reconstructions: (1) a GS reconstruction is applied to the low-pass Gaussian filtered input holograms and (2) a GA reconstruction is performed directly on the unfiltered input holograms. Then, the obtained GA result undergoes high-pass filtering using the same Gaussian kernel as employed in the earlier low-pass filtering step. The final reconstruction is produced by combining the low-pass filtered GS result and the high-pass filtered GA result. This merging step balances the shot noise averaging property provided by the GA method with the robust phase retrieval of the GS method. The combination ensures that both low- and high-frequency sample information is accurately reconstructed while minimizing noise artifacts.

Algorithm S4 – IGA method

Inputs: $H_n, \lambda_n, z_n, T, \sigma \quad (n = 1,2, \dots, N)$
Output: R

1. **for** $n = 1{:}N$
2. $H_n{}' = H_n * G(\sigma)$ % Low-pass filter input holograms
3. **end**
4. $R_{GS} = \mathrm{GS}(H'_{1:N}, \lambda_{1:N}, z_{1:N}, T)$ % GS reconstruction
5. $R_{GA} = \mathrm{GA}(H_{1:N}, \lambda_{1:N}, z_{1:N})$ % GA reconstruction
6. $Re(R'_{GA}) = Re(R_{GA}) - Re(R_{GA}) * G(\sigma)$ % High-pass filter real part of GA
7. $Im(R'_{GA}) = Im(R_{GA}) - Im(R_{GA}) * G(\sigma)$ % High-pass filter imaginary part of GA
8. $Re(R) = Re(R_{GS}) + Re(R'_{GA})$ % Combine real parts of GS and GA'
9. $Im(R) = Im(R_{GS}) + Im(R'_{GA})$ % Combine imaginary parts of GS and GA'
10. $R = Re(R) + i \cdot Im(R)$ % Final result

Fig. S4. The workflow of proposed iterative Gabor averaging method. $G(\sigma)$ represent a Gaussian kernel with given standard deviation (σ) and $*$ denotes the 2D convolution operation.

5. Influence of σ parameter

The performance of IGA algorithm is influenced by the choice the σ parameter, which controls the balance between the GA and GS components of the reconstruction process. Specifically, the value of σ dictates the relative contribution of each method: larger σ values increase the influence of the GA component, while smaller σ values emphasize the GS component.

Figure S5 illustrates the effect of varying σ on simulated phase reconstruction accuracy (simulation parameters are the same as for results shown in manuscript Fig. 4). The top panel of Fig. S5 shows the SSIM measure of the phase reconstructions as a function of input holograms SNR. The bottom panel displays exemplary reconstructions for both noise-free and noisy data. These results demonstrate a clear trade-off: small σ values (e.g., 0 and 0.5) tend to amplify shot noise in noisy datasets, leading to degraded reconstructions, whereas larger σ values (e.g., 4 and especially ∞) fail to sufficiently eliminate twin-image artifacts, a common limitation in Gabor-based reconstructions.

Optimal performance is achieved with σ values in the range of 1 to 4, where the twin-image artifacts are significantly reduced, without substantial amplification of shot noise. This balance allows the IGA algorithm to merge the strengths of GA's robust averaging with GS's superior twin image suppression, achieving high reconstruction fidelity even in the presence of significant noise.

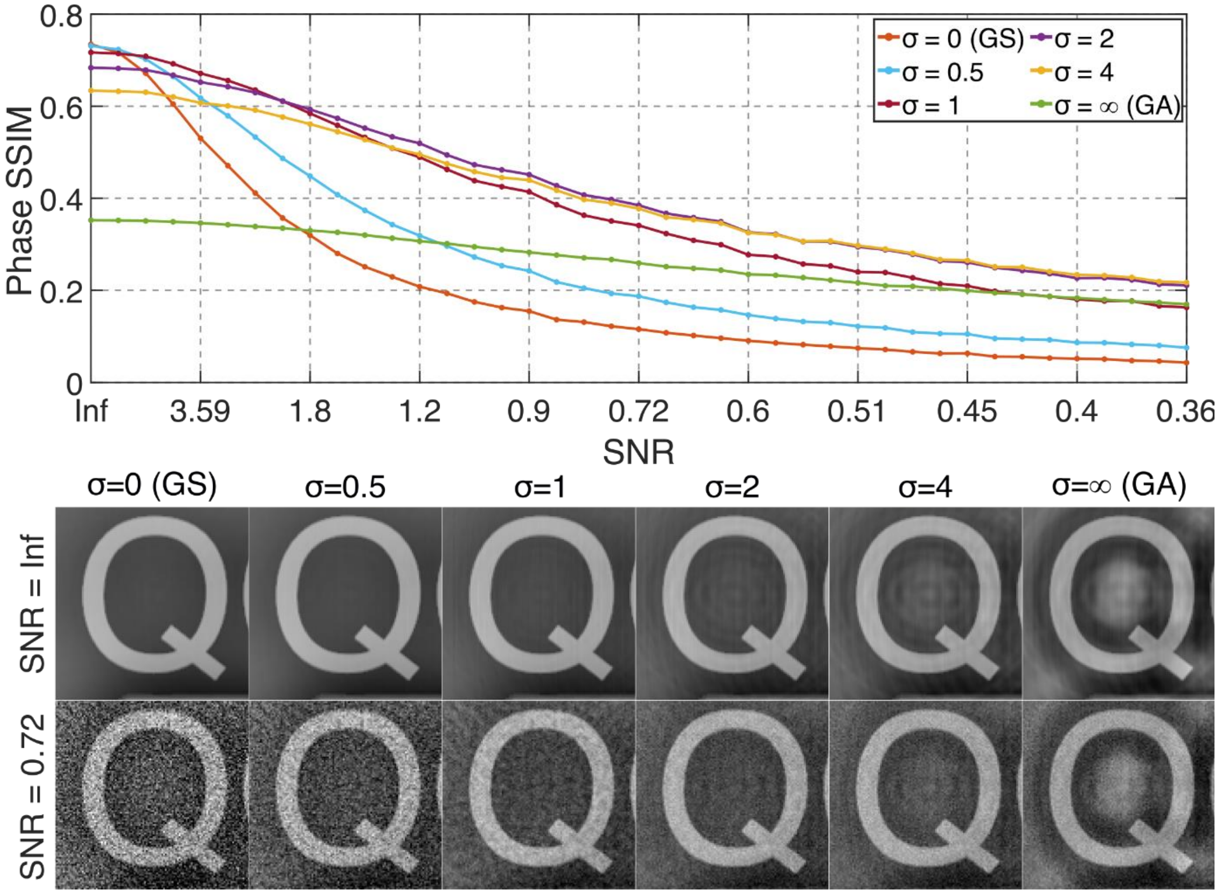

Fig. S5. Results of simulations from manuscript Fig. 4 for different σ value of IGA algorithm. Top – reconstructed phase SSIM in function of holograms SNR. Bottom – exemplifying reconstructions for shot noise-free and shot noise-spoiled data.

Figure S6 further explores the influence of σ by presenting reconstructions for both high photon budget (HPB) and low photon budget (LPB2) datasets, as described in manuscript Fig. 5. The results, assessed both qualitatively and quantitatively (via the background std and object SSIM), reaffirm the earlier observations: larger σ values are effective at averaging shot noise, while smaller work better with minimizing twin image artifacts. At σ = 1 or 2, the twin image suppression rivals that of the GS method, while shot noise is most effectively averaged for σ = 2 and 4. Based on these insights, σ = 2 was selected for all datasets processed in the manuscript.

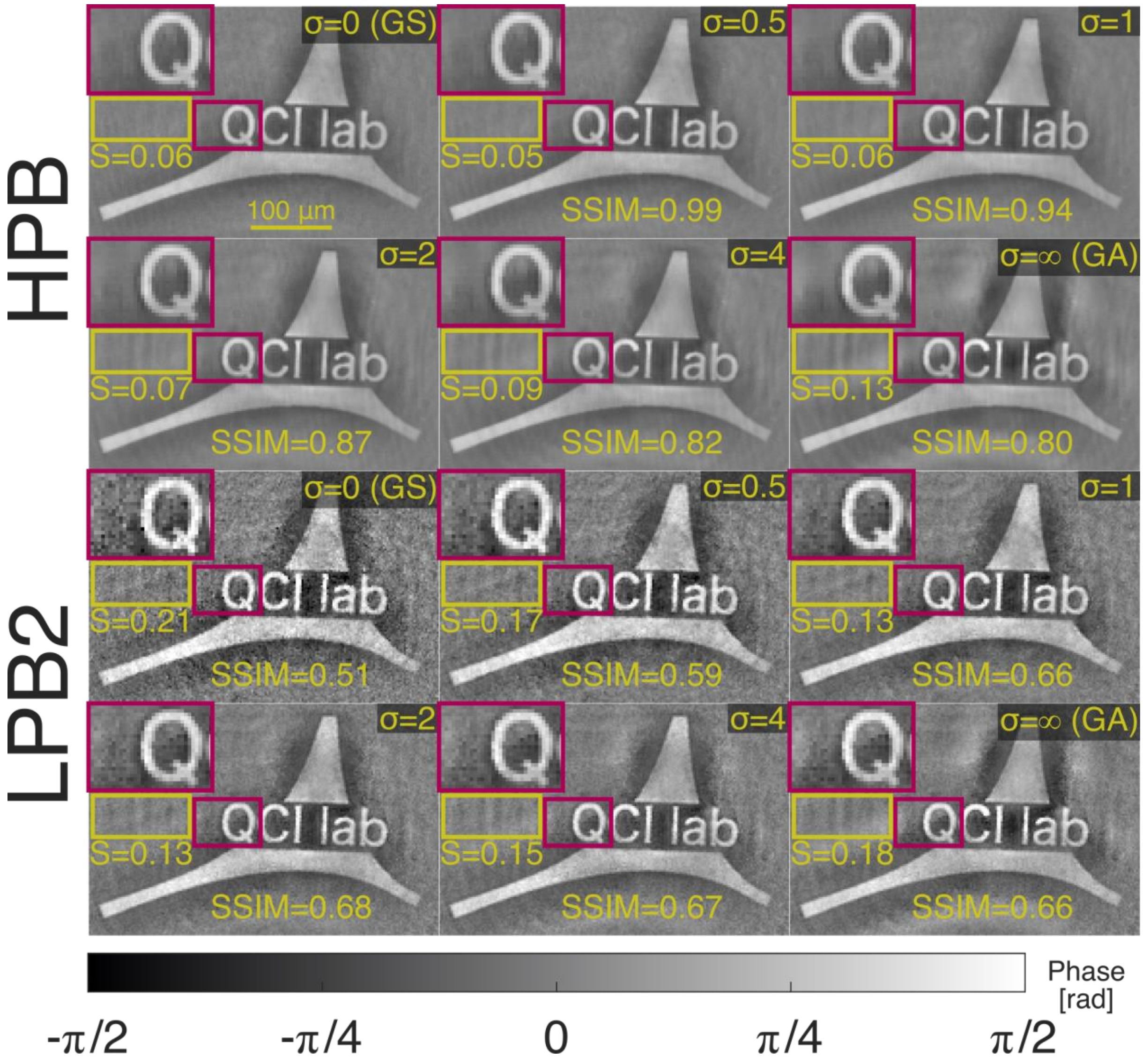

Fig. S6. IGA algorithm performance for different σ parameters for HPB and LPB2 data presented in manuscript Fig. 5. S – standard deviation of object-free area marked with yellow rectangle given in [rad] units.

6. Two-photon polymerization printed target

Custom-made phase test target (shown in manuscript Fig. 5) was fabricated using two-photon polymerization (TPP) technique. TPP allows a selective photo-polymerization curing (also called printing) of liquid resin, achieving nano-scale precision taking advantage of two-photon absorption phenomenon.[10] In this study we used commercial TPP setup - Photonic Professional GT2 from Nanoscribe GmbH. The setup was equipped with 25x, NA 0.8 immersion microscope using galvanometric scanning for fabrication movement. Polymeric IP-S resin (Nanoscribe GmbH)

was used in the fabrication process, achieving refractive index (RI) values in the range of 1.486 when liquid to 1.515 when fully polymerized.

As proposed in recent studies,[11–13] by altering monomer cross-linking during the polymerization, we achieved varying RI in fabricated structure by partial polymerization achieved manipulating laser dose power. Within physical properties of utilized resin, we were able to fabricate test structure with changing RI in the range of 1.495 – 1.515. The design preparation process included transcription of grayscale image (Fig. S7(a)) intensity values into laser power variation and then elongation of 2D image along Z-axis into 3D structure. The mentioned processing was performed in Matlab environment, as well as preparation of the direct printing code. Figure S1(b) displays schematic visualization of 3D printing file compiled in DeScribe software provided by Nanoscribe GmbH.

The fabricated structure was rinsed out of unpolymerized liquid remnant resin in isopropyl alcohol bath for 7 minutes and dried in the air. Target printed on ITO-coated soda lime glass substrate (25 x 25 x 0.7 mm^3) was immersed in 1.480 imaging immersion oil and covered with 170 µm thick cover glass. The distance between the substrate and the cover glass was kept by 120 µm thick imaging spacer. As the designed thickness of the test target was 3 µm, phase delay introduced by the structure should reach values in the range Δ = 1.2 rad.

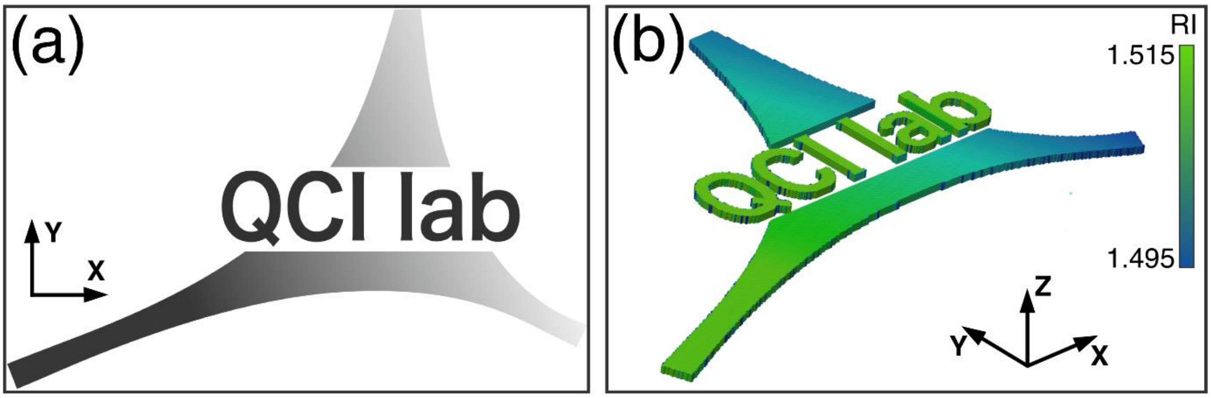

Fig. S7. (a) Input 2D grayscale image. (b) Visualization of 3D printed structure design in respect to refractive index value.

7. Human spermatozoa sample

A human sperm biosample was donated for the experiments by an anonymous subject. After conventional refrigerated transport (less than 12 h) and dose homogenization, 1 mL was placed in an Eppendorf tube, maintained for 20 min at 37 °C. Next, the semen sample was inserted by pipetting into a commercially available counting chamber. Neither pre-filtering nor pre-preparation (centrifugation, dilution, re-suspension, etc.) was applied, so the sample contained a significant amount of additional seminal particles. Roughly, the sperm cells have approximately a head length and width of 4 and 5 µm, respectively, a total length of 45 µm and a tail width below 1 µm. After the measurement, acquired in-line holograms were additionally preprocessed by subtraction of averaged frame (from a 300-frame video) in order to digitally remove the sample contaminations and immobile sperm cells.

Bibliography

(1) Matsushima, K.; Shimobaba, T. Band-Limited Angular Spectrum Method for Numerical Simulation of Free-Space Propagation in Far and near Fields. *Opt. Express* **2009**, *17* (22), 19662. https://doi.org/10.1364/OE.17.019662.

(2) Latychevskaia, T.; Fink, H.-W. Practical Algorithms for Simulation and Reconstruction of Digital In-Line Holograms. *Appl. Opt.* **2015**, *54* (9), 2424. https://doi.org/10.1364/AO.54.002424.

(3) Gerchberg, R. W. A Practical Algorithm for the Determination of Phase from Image and Diffraction Plane Pictures. *Optik (Stuttg).* **1972**, *35*, 237–246.

(4) Fienup, J. R. Phase Retrieval Algorithms: A Comparison. *Appl. Opt.* **1982**, *21* (15), 2758. https://doi.org/10.1364/AO.21.002758.

(5) Greenbaum, A.; Ozcan, A. Maskless Imaging of Dense Samples Using Pixel Super-Resolution Based Multi-Height Lensfree on-Chip Microscopy. *Opt. Express* **2012**, *20* (3), 3129. https://doi.org/10.1364/OE.20.003129.

(6) Greenbaum, A.; Zhang, Y.; Feizi, A.; Chung, P.-L.; Luo, W.; Kandukuri, S. R.; Ozcan, A. Wide-Field Computational Imaging of Pathology Slides Using Lens-Free on-Chip Microscopy. *Sci. Transl. Med.* **2014**, *6* (267), AW4J.6. https://doi.org/10.1126/scitranslmed.3009850.

(7) Zuo, C.; Sun, J.; Chen, Q.; Hu, Y.; Zhang, J.; Hu, Y.; Chen, Q. Lensless Phase Microscopy and Diffraction Tomography with Multi-Angle and Multi-Wavelength Illuminations Using a LED Matrix. *Opt. Express* **2015**, *23* (11), 14314. https://doi.org/10.1364/OE.23.014314.

(8) Gao, Y.; Cao, L. Projected Refractive Index Framework for Multi-Wavelength Phase Retrieval. *Opt. Lett.* **2022**, *47* (22), 5965. https://doi.org/10.1364/OL.476707.

(9) Micó, V.; Rogalski, M.; Picazo-Bueno, J. Á.; Trusiak, M. Single-Shot Wavelength-Multiplexed Phase Microscopy under Gabor Regime in a Regular Microscope Embodiment. *Sci. Rep.* **2023**, *13* (1), 4257. https://doi.org/10.1038/s41598-023-31300-9.

(10) Zhou, X.; Hou, Y.; Lin, J. A Review on the Processing Accuracy of Two-Photon Polymerization. *AIP Adv.* **2015**, *5* (3). https://doi.org/10.1063/1.4916886.

(11) Wdowiak, E.; Ziemczonok, M.; Martinez-Carranza, J.; Kuś, A. Phase-Assisted Multi-Material Two-Photon Polymerization for Extended Refractive Index Range. *Addit. Manuf.* **2023**, *73* (June), 103666. https://doi.org/10.1016/j.addma.2023.103666.

(12) Ziemczonok, M.; Kuś, A.; Wasylczyk, P.; Kujawińska, M. 3D-Printed Biological Cell Phantom for Testing 3D Quantitative Phase Imaging Systems. *Sci. Rep.* **2019**, *9* (1), 18872. https://doi.org/10.1038/s41598-019-55330-4.

(13) Schmid, M.; Ludescher, D.; Giessen, H. Optical Properties of Photoresists for Femtosecond 3D Printing: Refractive Index, Extinction, Luminescence-Dose Dependence, Aging, Heat Treatment and Comparison between 1-Photon and 2-Photon Exposure. *Opt. Mater. Express* **2019**, *9* (12), 4564. https://doi.org/10.1364/OME.9.004564.
